# Mcl-1 expression influcences CD8+ anti-tumor immunity.

**DOI:** 10.1186/2051-1426-3-S2-P323

**Published:** 2015-11-04

**Authors:** Lukas W Pfannenstiel, Brian R Gastman

**Affiliations:** 1 Cleveland Clinic, Cleveland, OH, USA

## 

The recent clinical successes of immunotherapies such as anti-PD-1 and anti-CTLA-4 antibody blockade have re-invigorated the idea that the immune system has the potential to be a powerful tool to destroy tumors. However despite ample evidence that these therapies are able to enhance the efficacy of anti-tumor CD8+ T cell responses, the association of pro-survival molecules such as Mcl-1 with this process remains poorly understood. We have previously shown that short-term *in vitro* co-culture of human T cells with human-derived tumor cell lines of various origins can induce the gain of senescence-like features CD8+ T cells, particularly the loss of CD27/CD28 expression and the gain of a potent suppressive function in *in vitro* suppression assays. In subsequent studies, we found that IL-7 could protect T cells from the development of dysfunction/suppression, and that this process is highly dependent on the expression of the pro-survival protein Mcl-1. In the current study we sought to determine whether the beneficial effects of IL-7 could also be shown *in vivo* in a mouse model of head and neck squamous cell carcinoma (HNSCC). We show that the use of exogenous IL-7 treatment results in a delay in tumor growth, and that the combination of IL-7 with other immunotherapies, particularly anti-PD-1 antibody blockade, results in a synergistic response which is better than either therapy alone. Further, when combined with an adoptive transfer of TCR transgenic T cells, IL-7 treatment results in enhanced delay of tumor growth and greater numbers of tumor-specific T cells in the tumor microenvironment. Similar to our previous *in vitro* studies, we further find that expression of Mcl-1 can have a significant effect on anti-tumor CD8+ T cells responses. Knock-down of Mcl-1 expression significantly abrogates the function of tumor-specific, adoptive-transferred T cells while the enhancement of Mcl-1 expression also enhances CD8+ function and significantly delays tumor growth. These data indicate that one benefit of existing immunotherapy strategies like IL-7 could be in the enhancement of expression of pro-survival factors like Mcl-1, and those future treatment strategies which specifically target their expression could improve anti-tumor immune responses.

